# Supplementary material for: Aspirin desensitization in NSAID-exacerbated respiratory disease and its outcomes in the clinical course of asthma: A systematic review of the literature and meta-analysis
Source: PLoS One. 2021 Mar 26;16(3):e0247871. doi: 10.1371/journal.pone.0247871 (PMC7996991; doi:10.1371/journal.pone.0247871)
Supplement: S1 File — (DOCX) [file pone.0247871.s003.docx]

**Aspirin desensitization in NSAID-exacerbated respiratory disease and its outcomes in the clinical course of asthma: A systematic review of the literature and meta-analysis**

**Introduction**

**Background**

**Description of the condition**

Non-steroidal anti-inflammatory drugs (NSAID) - exacerbated respiratory disease (NERD) was described 50 years ago by Samter and Beers, previously known as aspirin-induced asthma or aspirin-exacerbated respiratory disease (AERD) [1]; there is a chronic eosinophilic inflammation of the respiratory tract, accompanied by nasal polyps, chronic rhinosinusitis and/or asthma, and the symptoms are typically exacerbated by NSAIDs, including aspirin (ASA) [1, 2]. NERD requires follow-up by several specialties: pulmonology to manage difficult to control asthma, allergology for the management of hypersensitivity to NSAIDs and chronic eosinophilic inflammation, and otolaryngology due to the recurrence of nasal polyps and requirement of surgery [1].

The prevalence of NERD varies from 1.8-44% depending on the study population and the diagnostic criteria used [1]; in the general population, it lies around 0.3-0.9%, among asthmatic patients 10-20%, severe asthma 15%, 24% in patients admitted to intensive care unit for asthma exacerbation and 30-40% in asthmatics with concurrent nasal polyps, increasing in parallel to the severity of the disease [1,2]. It affects 1 in 10 adults with asthma or chronic rhinosinusitis and nasal polyps [3]. Furthermore, aspirin hypersensitivity has been proposed in the literature as an independent risk factor for developing severe asthma [4]. Patients with NERD have twice the risk of having uncontrolled asthma, 60% more asthma exacerbations, 80% more emergency consultations, and 40% more hospitalizations. Additionally, they require more asthma medications and have a poorer quality of life when compared to patients without NERD [9-11].

Among the risk factors to develop NERD, a family history of the disease, presence of nasal polyps associated with chronic rhinosinusitis and/or asthma, and atopy stand out, alongside a slight predisposition of female patients as compared to male population [1, 5, 6]. Also, certain epigenetic polymorphisms may lead to an increased risk of developing NERD [7]. The disease is usually diagnosed in the 3^rd^ - 4^th^ decade of life and its natural history involves chronic rhinitis as the first manifestation, progressing to chronic rhinosinusitis, nasal polyps, and anosmia. During the latter period, asthma appears to be triggered [5], and often happens before acquiring hypersensitivity to NSAIDs. However, there are cases in which hypersensitivity to NSAIDs occurs before the onset of chronic airway disease [1]. Despite NSAID avoidance, patients continue to have asthma exacerbations, loss of smell, and the need for multiple sinus surgeries [8].

In patients with NERD, there is an overexpression of Th2 mediators such as IL 33, thymic stromal lymphopoietin (TSLP), which allows the attraction of eosinophils. The exacerbation of respiratory symptoms by NSAIDs is due to their ability to block the production of prostaglandin E2 (PGE2) by inhibiting cyclooxygenase 1 (COX-1); the deficiency of this anti-inflammatory mediator is not compensated by the production of prostaglandin D2 (PGD2) through the cyclooxygenase 2 (COX-2) pathway, so the excess of arachidonic acid is metabolized by 5 lipooxygenase (5 LO) generating the accumulation of powerful inflammatory mediators such as leukotrienes LTC4, LTD4, and LTE4. It has also been shown that patients with NERD have an abnormal function at PGE2 receptors [1].

After the intake of NSAIDs, symptoms appear within 30-180 minutes, the onset and severity of which is associated with the dose administered. Most patients develop symptoms with 60 mg of acetylsalicylic acid (ASA), but this range varies from 10-300 mg [1, 15, 16]. Manifestations are characterized by high respiratory symptoms such as nasal congestion and rhinorrhea and may progress to wheezing, coughing, and dyspnea. In patients with uncontrolled asthma, symptoms appear more rapidly and severely, and could potentially lead to fatal outcomes [17]. Skin manifestations such as hives or rash, and gastrointestinal (GI) symptoms are also common [1, 6]. Less frequently, patients manifest symptoms associated with alcohol consumption, finding eosinophilia in the blood work [1].

The clinical history is key to making a diagnosis of NERD. The appearance of respiratory symptoms 1-2 hours after the consumption of NSAIDs, in patients with adult-onset asthma and with a history of repeated nasal polyposis are key to identifying patients with NERD. If the patient does not meet all the criteria or there is doubt in the diagnosis, NSAID hypersensitivity must be confirmed using an oral provocation test, in which increasing doses of the drug are administered following established protocols. This should be done in a safe environment with adequate staff and equipment to ensure an appropriate response to any reactions, such as conjunctivitis or rhinitis, low respiratory symptoms, bronchospasm, a decline in pulmonary function (a decrease in forced expiratory volume in one second (FEV1) by more than 15%), laryngospasm, cutaneous manifestations and systemic symptoms [1, 16]. Oral provocation tests are contraindicated in patients with previous anaphylactic reactions associated with NSAIDs or ASA, uncontrolled asthma with FEV1 <70% of the predictive value, history of chronic renal failure or gastrointestinal bleeding, an exacerbation of asthma in the previous month, pregnancy and present management with beta-blockers [1].

The mainstay in management is the avoidance of the causative drug and other strong COX-1 inhibitor molecules; typically, NERD patients tolerate selective COX-2 inhibitors such as celecoxib and etoricoxib. It is of utmost importance that patients are educated about their disease, know about alternative medications that are safe for them, and avoid alcohol consumption as it can worsen symptoms [1). Specifically, for the asthmatic population, their treatment is done according to various guidelines developed by The National Heart, Lung, and Blood institute (NHLBI), The Global Initiative for Asthma (GINA), and The British Thoracic Society (BTS), among others [18]. It is reported that 80% of patients with NERD require management with high doses of inhaled steroids and 51% need oral corticosteroids [2]. Also, these patients usually benefit from management with leukotriene antagonists to try to reduce the existing over-expression of cysteinyl leukotrienes and in particular cases, they require management with biologics such as Omalizumab or anti IL5 molecules [1, 19]. Concerning the management of chronic rhinosinusitis and nasal polyps, patients with NERD are more resistant to the usual pharmacological treatments, such as intranasal steroids, also requiring oral steroids for the control of the disease. These patients frequently require multiple surgical reinterventions for recurrence of nasal polyposis (ranging from 24-80% of patients), every 3 years approximately [1, 2].

**Description of the intervention**

The use of aspirin desensitization (AD) was initiated in 1922 by Widal et al., who also described the oral provocation test to aspirin in patients with NERD. In 1980, Stevenson et al. reported a decrease in nasal symptom frequency, fewer hospitalizations, and less use of systemic steroids in patients with NERD following AD [20]. The desensitization process aims to generate tolerance to ASA, existing different protocols for performing it, but all include the administration of ascending doses of ASA at intervals of 90-120 minutes until a reaction or the target dose is reached within 1-3 days. If a reaction occurs before achieving the target dose, the process must start again the next day (table 1). During the process, drug-induced reactions become milder and shorter until they disappear [1,6]. After completing the AD patients must continue to receive a daily dose of ASA, ranging from 300-1300 mg/day, during a prolonged period to avoid loss of tolerance to aspirin [1, 2, 16] (table 1).

| Table 1. Aspirin desensitization protocol (Table extracted from: Kowalski ML, Agache I, Bavbek S, et al. Diagnosis and management of NSAID-Exacerbated Respiratory Disease (N-ERD)—a EAACI position paper. Allergy Eur J Allergy Clin Immunol. 2019) | | |
| --- | --- | --- |
| Time | Day 1 | Day 2 |
| 9:00 AM | 20-40 mg | 100- 160 mg |
| 11:00 AM | 40-60 mg | 160-325 mg |
| 01:00 PM | 60-100 mg | 325 mg |

While performing AD, ocular, nasal, bronchial, laryngeal, cutaneous and GI symptoms may occur, equal to those triggered by the oral provocation test; therefore, AD should also be performed with caution, following established protocols, in safe environments and with trained personnel [1, 16]. A severe reaction associated with NSAIDs is not considered a contraindication for AD, since the severity of previous reactions does not predict future ones [15]. Also, it is recommended that patients are taken for sinus cytoreduction 2 to 4 weeks before AD because desensitization has not been shown to have an impact on polyp size [16].

Given the long-term administration of aspirin, patients may present with 2 common adverse effects during the desensitization process: gastric ulcers secondary to decreased prostaglandin I2 synthesis and inadequate repopulation of gastric mucosal cells in <15% of patients, and bleeding, which occurs predominantly in the skin but can also occur in the nose, bronchi or GI tract [6].

**How the intervention might work**

It is known that in NERD there is a deregulation of the inflammatory and anti-inflammatory mediators produced by the metabolism of arachidonic acid causing: an elevated expression of cysteine leukotriene receptors (CysLT), a concomitant increase in mast cells and eosinophils in the tissues and a decrease in the synthesis of PGE2 which functions as an inhibitor of 5-LO and leukotriene production [20-22]. Associated with this is the description that the alteration in the inflammatory mediators in NERD is related to the overexpression of IL4 that triggers the activation of leukotrienes [20].

It has been suggested that AD followed by maintenance of a daily dose of aspirin improves deregulation of arachidonic acid metabolism by reducing activation of tyrosine kinase, generating inhibition of STAT6 phosphorylation, which leads to a decrease in IL4 production with downward regulation of CysLT production and reduced expression of the CysLT1 receptor, which ultimately leads to attenuation of airway inflammation and clinical improvement [23]. Besides, patients with AD followed by daily dose have been shown to decrease urinary PGD2 levels, which may be related to decreased effector cell chemotaxis within the tissues, since PGD2 is a potent chemotherapeutic factor for TH2 cells and contributes to a large extent to the eosinophilic inflammation observed in patients with NERD [20].

Patients with NERD who benefit from AD followed by the administration of a daily dose of aspirin are: patients with moderate-severe asthma, with inadequate control of nasal symptoms, who show little response to pharmacological management, recurrence of nasal polyps, need for systemic corticosteroids for the control of NERD, prevention of nasal polyps after surgery or patients who require aspirin for another condition such as coronary ischemic disease or chronic anti-inflammatory management [1, 2].

Desensitization to aspirin has shown multiple benefits: improved quality of life, reduced symptoms of high congestion, improved smell, decreased polyp formation and need for surgery, decreased use of systemic corticosteroids, and improved asthma control in patients with NERD [2, 16, 24].

Since patients with NERD present difficulties in the management of both asthma and chronic rhinosinusitis, desensitization to ASA is proposed as an option to improve the course of chronic rhinosinusitis and asthma in these patients.

**Why it is important to do this review**

Oral provocation testing and AD followed by daily ASA therapy are important tools for both diagnosis and specific treatment of NERD, which offers clinical benefit to patients [1, 6]. The goals of NERD management are to decrease inflammation in the upper and lower airway allowing the prevention of nasal polyp formation, secondary sinusitis, and asthma exacerbation. In patients in whom this objective is not achieved with the usual pharmacological treatment or requires continuous oral steroid doses, desensitization is proposed as a therapeutic option for the control of upper and lower respiratory tract symptoms.

Systematic reviews focused on evaluating the efficacy of AD in improving nasosinusal symptoms have been conducted and found to be a valuable adjunct in the management of these patients [25, 26], but there are no reviews focused on evaluating the effect that AD has on the clinical course of asthma focusing on changes in lung function, decreased steroid use and quality of life in patients with asthma. Therefore, there is a need to conduct this review based on the studies published to date, to clarify the effects of AD in patients with NERD in terms of outcomes in the clinical course of asthma, given that this may be an important therapeutic option in these patients, being more affordable when compared to currently available biological drugs [2, 6].

**Objectives**

To analyze the clinical effects of AD compared to placebo in terms of lung function, systemic and inhaled steroid use, frequency of acute asthma exacerbations, and adverse effects, in patients with NERD and asthma.

**Primary objectives**

1. Analyze the efficacy of AD regarding improvement of lung function
2. Analyze the effect of AD in reducing systemic steroid use
3. Analyze the effect of AD in reducing inhaled systemic steroid use
4. Determine the effect of AD in frequency of asthma acute exacerbations

**Secondary objectives**

1. Analyze the effect of AD in the quality of life of patients with NERD
2. Determine the frequency of adverse effects associated to AD.

**METHODS**

**Criteria for considering studies for this review**

**Types of studies**

For this review, we will include published randomized clinical trials (RCTs) with a parallel design. We will not exclude non blinded studies nor randomized pilot studies, but we open-label trials will be excluded due to the high risk of bias of non-randomized studies- Studies should have a minimum of 3 months follow-up. We will consider manuscripts in Spanish and English.

**Type of participants**

**Inclusion criteria**

Patients ≥ 18 years-old with a diagnosis of asthma, associated with chronic rhinosinusitis and nasal polyps, with a previous history of pulmonary symptoms triggered by ASA or other NSAID, or with a positive provocation test to ASA.

**Exclusion criteria**

Patients with a history of GI bleeding, bleeding diathesis, uncontrolled arterial hypertension, chronic renal failure, uncontrolled asthma with FEV1 < 70%, autoimmune disorders, malignancy, or pregnancy.

Patients with a history of other pulmonary diseases, such as cystic fibrosis or primary ciliary dyskinesia.

**Types of outcome measures:**

**Primary outcomes:**

1. Change in FEV1 and FEV1/FVC during spirometry
2. Frequency and/or total dosing of systemic steroids
3. Frequency and/ or total dosing of inhaled steroids
4. Frequency of acute asthma exacerbations

**Secondary outcomes:**

1. Change in quality of life using asthma control test (ACT)/visual analogue scale (VAS) or symptom score.
2. Frequency of adverse events

**Search methods for identification of studies**

**Electronic searches**

A Pubmed, EMBASE, SCOPUS, EBSCO search will be performed using controlled vocabulary (Mesh, Emtree terms) as well as free text with the following search terms: aspirin exacerbated respiratory disease, aspirin desensitization, acetyl salicylic acid, asthma.

The results will be limited to human based clinical trials, written in English or Spanish. References will be manually searched for additional relevant studies.

**Data collection and analysis**

**Selection of studies**

Studies will be screened by two independent investigators^IE, SS^, who will analyze the titles and abstracts for potentially relevant studies. Then each study will be assessed against the preset inclusion and exclusion criteria.

**Data extraction and management**

Two investigators^IE, SS^ will extract the data from all studies using a standardized data collection form. When disagreements are present, a third party will be consulted. For each study, the trial design, characteristics of participants, type of interventions and outcomes will be assessed. We will use the Cochrane Review Manager (RevMan 5.1) software to analyze data.[12]

**Assessment of risk of bias in the included studies**

Bias will be assessed according to the recommendations outlined in the Cochrane Handbook for Systematic Reviews of Interventions.[13] The following items will be analyzed:

- Allocation sequence generation;
- Concealment of allocation;
- Blinding of participants and investigators;
- Incomplete outcome data;
- Selective outcome reporting.

We will grade each potential source of bias as low risk, high risk or unclear risk of bias.

**Measures of treatment effect**

**Dichotomous data**

We will analyze dichotomous data variables using Mantel-Haenzsel odds ratios using a fixed-effect model with 95% confidence intervals. If substantial heterogeneity is found among the studies a random effect model will be chosen. If count data is not reported as the number of events per participant, the variable will be transformed into a continuous one.

**Continuous data**

Continuous variables will be analyzed as fixed effect mean differences with 95% confidence intervals. If substantial heterogeneity is found among the studies a random effect model will be chosen. Data will be collected using Intention to treat (ITT) analysis when possible.

**Dealing with missing data**

We will not contact authors for missing data but we will take this into consideration when judging the quality of evidence and when analyzing the results.

**Assessment of heterogeneity**

We will evaluate the degree of statistical variation using the I^2^ statistic.

**Assessment of reporting biases**

We will compare the outcomes reported in the methods sections of the articles with the published results to check for reporting biases. We Furthermore, we plan to do funnel plots when possible.

**Data synthesis**

We will present the findings of our primary outcomes in the “Summary of findings table” produced using the GradePro software.[14]

**References**

1. Kowalski ML, Agache I, Bavbek S, Bakirtas A, Blanca M, Bochenek G, et al. Diagnosis and management of NSAID-Exacerbated Respiratory Disease (N-ERD)—a EAACI position paper. Allergy Eur J Allergy Clin Immunol. 2019;74(1):28–39.

2. Comert S, Celebioglu E, Yucel T, Erdogan T, Karakaya G, Onerci M, et al. Aspirin 300 mg/day is effective for treating aspirin-exacerbated respiratory disease. Allergy Eur J Allergy Clin Immunol. 2013;68(11):1443–51.

3. Rajan. JP, Wineinger. NE, Stevenson. DD WA. Prevalence of aspirin‐exacerbated respiratory disease among asthmatic patients: a meta‐analysis of the literature. J Allergy Clin Immunol. 2015;135:676–81.

4. European Network for Understanding Mechanisms of Severe Asthma. The ENFUMOSA cross‐sectional European multicentre study of the clinical phenotype of chronic severe asthma. Eur Respir J. 2003;22:470‐477.

5. Fahrenholz JM. Natural history and clinical features of aspirin-exacerbated respiratory disease. Clin Rev Allergy Immunol. 2003;24(2):113–24.

6. White AA, Stevenson DD. Aspirin-exacerbated respiratory disease. N Engl J Med. 2018;379(11):1060–70.

7. Kim. SH, Sanak. M PH. Genetics of hypersensitivity to aspirin and nonsteroidal anti‐inflammatory drugs. Immunol Allergy Clin North Am. 2013;33:177‐194.

8. Szczeklik. A, Nizankowska. E DM. Natural history of aspirin induced asthma. AIANE Investigators. European Network on Aspirin Induced Asthma. Eur Respir J. 2000;16:432‐436.

9. Schatz. M, Hsu. JW, Zeiger. RS et al. Phenotypes determined by cluster analysis in severe or difficult‐to‐treat asthma. J Allergy Clin Immunol. 2014;133:1549‐1556.

10. Respiratory hypersensitivity reactions to NSAIDs in Europe: the global allergy and asthma network (GALEN). Allergy. 2016;71:1603‐1611.

11. Morales. DR, Guthrie. B, Lipworth. BJ, Jackson. C, Donnan. PT, Santiago. VH. NSAID‐exacerbated respiratory disease: a meta‐analysis evaluating prevalence, mean provocative dose of aspirin and increased asthma morbidity. Allergy. 2015;70:828‐835.

12. Stevens. WW, Peters. AT, Hirsch. AG et al. Clinical characteristics of patients with chronic rhinosinusitis with nasal polyps, asthma, and aspirin‐exacerbated respiratory disease. J Allergy Clin Immunol Pr. 2017;5:1061‐1070.

13. Kowalski. ML, Bienkiewicz. B, Pawliczak. R KP. Nasal polyposis in aspirin‐hypersensitive patients with asthma (aspirin triad) and aspirin‐tolerant patients. Allergy Clin Immunol Int ‐ J World Allergy Org. 2003;6:246‐250.

14. Mullol. J PC. Rhinosinusitis and nasal polyps in aspirin‐exacerbated respiratory disease. Immunol Allergy Clin North Am. 2013;33:163‐176.

15. Hope. AP, Woessner. KA, Simon. RA SD. Rational approach to aspirin dosing during oral challenges and desensitization of patients with aspirin‐exacerbated respiratory disease. J Allergy Clin Immunol. 2009;123:406‐410.

16. Waldram JD, Simon RA. Performing Aspirin Desensitization in Aspirin-Exacerbated Respiratory Disease. Immunol Allergy Clin North Am [Internet]. 2016;36(4):693–703. Available from: http://dx.doi.org/10.1016/j.iac.2016.06.006

17. Yoshimine. F, Hasegawa. T, Suzuki. E et al. Contribution of aspirinin tolerant asthma to near fatal asthma based on a questionnaire survey in Niigata Prefecture, Japan. Respirology. 2005;10:477‐484.

18. Global Strategy for Asthma Management and Prevention. GINA Report, Global Strategy for Asthma Management and Prevention. 2020;

19. Berges-Gimeno. MP, Simon. RA SD. The effect of leukotriene‐modifier drugs on aspirin‐induced asthma and rhinitis reactions. Clin Exp Allergy. 2002;32:1491‐1496.

20. Hill J, Burnett T, Katial R. Mechanisms of Benefit with Aspirin Therapy in Aspirin-Exacerbated Respiratory Disease. Immunol Allergy Clin North Am [Internet]. 2016;36(4):735–47. Available from: http://dx.doi.org/10.1016/j.iac.2016.06.011

21. Daffern PJ, Muilenburg D, Hugli TE SD. Association of urinary leukotriene E4 excretion during aspirin challenges with severity of respiratory responses. J Allergy Clin Immunol. 1999;104(3 Pt 1):559‐564.

22. Szczeklik A SD. Aspirin-induced asthma: advances in pathogenesis, diagnosis, and management. J Allergy Clin Immunol. 2003;111(5):913‐92.

23. Burnett T, Katial RK AR. Mechanisms of aspirin desensitization. Immunol Allergy Clin North Am. 2013;33(2):23.

24. Berges-Gimeno. M, Simon. RA SD. Long-term treatment with aspirin desensitization in asthmatic patients with aspirin-exacerbated respiratory disease. J Allergy Clin Immunol. 2003;111:180–186.

25. Larivée N, Chin CJ. Aspirin desensitization therapy in aspirin-exacerbated respiratory disease: a systematic review. Int Forum Allergy Rhinol. 2020;10(4):450–64.

26. Chu DK, Lee DJ, Lee KM, Schünemann HJ, Szczeklik W, Lee JM. Benefits and harms of aspirin desensitization for aspirin-exacerbated respiratory disease: a systematic review and meta-analysis. Int Forum Allergy Rhinol. 2019;9(12):1409–19.
