# Supplementary material for: Aspirin desensitization in NSAID-exacerbated respiratory disease and its outcomes in the clinical course of asthma: A systematic review of the literature and meta-analysis
Source: PLoS One. 2021 Mar 26;16(3):e0247871. doi: 10.1371/journal.pone.0247871 (PMC7996991; doi:10.1371/journal.pone.0247871)
Supplement: S1 Table — (DOCX) [file pone.0247871.s004.docx]

**S1 Table. Characteristics of Included Studies**

**Mortazavi 2017**

| **Methods** | Design: double blind randomized placebo-controlled study  Setting: 2 centers of immunology and allergy in Iran |
| --- | --- |
| **Participants** | Participants: n = 41 patients (Intervention: 22, placebo:19)  Baseline characteristics: mean age in intervention group 33 ± 2 years old, mean age in placebo group 29 ± 1 years old. Gender distribution 20 males:18 females.  FEV1 intervention group 74.68%±5.9, FEV1 placebo group 81.84% ± 7.8.  Diagnostic criteria: Aspirin challenge test with a decrease in FEV1 > 15% plus extrabronchial symptoms after aspirin administration  Inclusion criteria: > 18 years old, diagnosis of AERD.  Exclusion criteria: FEV1 < 70% in spirometry, history of anaphylactic or type 1 hypersensitivity reaction to aspirin, a history of gastrointestinal bleeding, bleeding incontinence, liver dysfunction, pregnant women. |
| **Interventions** | Intervention:   - Day 1: intranasal ketorolac spray at intervals of 30 min, followed by 2 dosages of 60 mg aspirin with an interval of 90 minutes. - Day 2: 160 mg aspirin, followed by 325 mg aspirin 180 min later. - Maintenance therapy: 650 mg aspirin BID for 1 month, then 325 mg aspirin BID for 4 months.   Other medications: standard asthma and nasal symptoms medications. |
| **Outcomes** | Primary outcomes: at 1- and 6-months quality of life (SNOT-22) and pulmonary function (FEV1).  Secondary outcomes: at 1- and 6- months IL4 and IL5. |
| **Notes** | No formal demographic and baseline clinical characteristics table (table 1) reported. |

***Risk of bias***

| **Bias** | **Authors judgment** | **Support for judgment** |
| --- | --- | --- |
| Random sequence generation (selection bias) | Low risk | Patients were randomly assigned in blocks using a random computer generator. Limited information on baseline characteristics. |
| Allocation concealment (selection bias) | Low risk | Aspirin and placebo capsules were prepared by a pharmacy student, who was not part of the study. |
| Blinding of participants and personnel (performance bias) | Low risk | Placebo capsules contained starch and were similar to aspirin. |
| Blinding of outcome assessment (detection bias) | Low risk | Study participants and investigators remained blinded until the study was completed |
| Incomplete outcome data (attrition bias) | Low risk | Attrition rate 7.4%, only 1 patient withdrawal was due to treatment adverse effects. |
| Selective reporting (reporting bias) | Low risk | All collected data was reported |

STEVENSON 1984

| **Methods** | Design: Double blind crossover study  Setting: USA General Clinical Research Center, Scripps Clinic and Research Foundation. |
| --- | --- |
| **Participants** | Participants: n = 38  Baseline characteristics: Mean age 45 years (18-71), Gender M:8 F:17, mean duration of respiratory tract disease 15 yr. 80% had nasal polyps. 72% had undergone sinus-drainage surgery in the past.  Diagnostic criteria: Patients who developed a bronchospastic reaction to ASA, with FEV, values declining 25% or more from baseline, and with FEV, values during placebo challenges changing <15%.  Inclusion criteria: Asthmatic patients with known or suspected ASA sensitivity.  Exclusion criteria: No information |
| **Interventions** | Intervention: Desensitization:  Day 1: oral ASA 3–600 mg until reaction  Day 2: dosing until 650 mg reached Maintenance: ASA (ASA 325 mg and Maalox 150 mg) or identical Ascriptin placebo tablets (Maalox 475 mg) daily × 3 months.  During the fourth month (washout period) study pills were discontinued.  At the end of the washout period, Study pills were then restarted but “crossed over” to the opposite drug for the last 3 mo of the study. |
| **Outcomes** | Primary outcomes: To test the hypothesis that ASA desensitization, followed by daily ingestion of ASA, improves the clinical course of rhinosinusitis or asthma in ASA sensitive subjects  Secondary outcomes: Decrease consumption of medications |
| **Notes** | 38 patients entered the double-blind crossover study. Of these 38 patients, 13 (34%) dropped out of the study for the following reasons: ASA Phase: 3 patients experienced gastrointestinal intolerance, 3 patients experienced increased asthma symptoms and 2 psychosocial reasons.  Placebo Phase: 1 patient experienced increased nasal congestion, 1 patient developed uterine bleeding, ‘and her surgeon requested discontinuation of the study to allow hysterectomy and 3 psychosocial reasons. |

Risk of bias

| **Bias** | **Authors judgment** | **Support for judgment** |
| --- | --- | --- |
| Random sequence generation (selection bias) | Low risk | Patient at entry into the study by use of a random  number system. |
| Allocation concealment (selection bias) | Low risk | Confidential records of dispensed Ascriptin and Ascriptin placebo were kept in the pharmacy, and the codes were broken only after the study was completed. |
| Blinding of participants and personnel (performance bias) | Low risk | Ascriptin tablets (ASA 325 mg and Maalox 150 mg) and identical Ascriptin placebo tablets (Maalox 475 mg) |
| Blinding of outcome assessment (detection bias) | Low risk | Study participants and investigators remained blinded to study drug until the study was completed. |
| Incomplete outcome data (attrition bias) | High risk | Withdrawal rate was considerate at 34%, of which 15.7% was due to adverse events. |
| Selective reporting (reporting bias) | Unclear risk | No protocol available, no information on pulmonary function which was stated as an outcome in the methods section. |

**Esmaeilzadeh 2015**

| **Methods** | Design: double blind randomized placebo-controlled study  Setting: Tehran University of Medical Sciences, Iran. |
| --- | --- |
| **Participants** | Participants: n = 34 patients (Intervention:18, placebo:16)  Baseline characteristics: mean age in intervention group 31 ± 4.3 years old, mean age in placebo group 27 ± 5.5 years old. Gender distribution: Intervention group 72% female, placebo group 62% female.  Lund Mackay score: intervention 15.1± 0.7, placebo group 12.4 ± 0.3.  Medication score: intervention group 13.1 ± 0.3, placebo group 11.5 ± 0.6.  Symptom score: intervention group 14.5 ± 1.1, placebo group 11.3 ± 0.9.  SNOT-22 score: intervention group 52.8 ± 4.1, placebo group 37.6 ± 2.7.  FEV1 intervention group 79.1%±1.9, FEV1 placebo group 83.8% ± 2.0.  Diagnostic criteria: aspirin challenge test for aspirin hypersensitivity in patients with asthma and chronic rhinosinusitis with nasal polyposis (CRSwNP). Test was considered positive if FEV1 decreased more than 20% of the baseline value or 15-19% plus naso-occular reactions, urticaria or angioedema.  Inclusion criteria: > 18 years old, CRSwNP with endoscopic and CT findings, stable asthma with no increase in systemic glucocorticoid for at least 3 months or no asthma attack in the last 6 months, aspirin hypersensitivity confirmed by a positive intranasal Ketorolac and modified oral aspirin challenge test.  Exclusion criteria: FEV1 < 70% in spirometry, smoking, pregnancy or current breastfeeding, history of bleeding diathesis and gastrointestinal bleeding, history of ischemic heart disease, stroke, diabetes, abnormal liver function and uncontrolled hypertension. |
| **Interventions** | Intervention:   - Run-in period: 3 months investigating asthma control and treatment compliance with monthly visits. - Day 1: 4 doses of intranasal ketorolac spray at intervals of 30 min, followed by 2 dosages of 60 mg aspirin with an interval of 90 minutes. - Day 2: 150 mg aspirin followed by 325 mg aspirin 180 min later. - Maintenance therapy: 650 mg aspirin BID for 1 month, then 325 mg aspirin BID for 6 months.   Other medications: standard asthma and medications for naso-ocular symptoms. |
| **Outcomes** | Primary outcomes: at 6 months sino-nasal-related quality of life (SNOT-22 score) and effect on anti-inflammatory cytokines (serum IL-10, TGF-B, IFN-Y).  Secondary outcomes: at 6 months Lund-MacKay score, symptom score, medication need score, FEV1. |
| **Notes** |  |

***Risk of bias***

| **Bias** | **Authors judgment** | **Support for judgment** |
| --- | --- | --- |
| Random sequence generation (selection bias) | Low risk | Patients were randomly assigned in blocks of 4 using a random computer generator. Limited information on baseline characteristics other than baseline outcome measures. |
| Allocation concealment (selection bias) | Unclear risk | Randomization list managed by unblinded study director |
| Blinding of participants and personnel (performance bias) | Low risk | Placebo:  Nasal spray with normal saline and capsules containing glucose which were similar to aspirin. |
| Blinding of outcome assessment (detection bias) | Low risk | Study participants and investigators remained blinded until the study was completed |
| Incomplete outcome data (attrition bias) | Low risk | Attrition rate 6%; 2 patients withdrew due to treatment adverse effects. |
| Selective reporting (reporting bias) | Low risk | All collected data was reported |

FRUTH 2013

| **Methods** | Design: double blind randomized placebo-controlled study  Setting: University Medical Center of the Johannes Gutenberg University Mainz, Langenbeckstrasse. |
| --- | --- |
| **Participants** | Participants: n = 70 patients  Baseline characteristics: Mean age 45 years SD 11years, 31 female  Diagnostic criteria: Chronic rhinosinusitis (CRS) was diagnosed by nasal endoscopy and by computer tomography  The underlying pathologic eicosanoid profile of AERD was confirmed by the functional in vitro test (LipiDOC-AIT, Erlangen, Germany).  Patients with an AIT value higher than 0.7 combined with recurrent nasal polyps and with the characteristic clinical symptoms were regarded as individuals suffering from AERD  Inclusion criteria: Individual with nasal polyps and AERD underwent sinus surgery at least twice  Exclusion criteria: Pregnant women and individuals with hemorrhagic diathesis, chronic gastric or duodenal ulcers, glucose-6-phosphate dehydrogenase deficiency, renal or liver diseases and individuals who took anticoagulant |
| **Interventions** | Intervention: Aspirin desensitization  Day 1: A cumulative aspirin dose of 180 mg  Day 2: A cumulative aspirin dose of 800 mg  Day 3: A maintenance dose from the third day on was set to 100 mg daily  Follow up: 6, 9, 12, 24, and 36 months |
| **Outcomes** | Primary outcomes:  Recurrence of nasal polyp  Secondary outcomes: Sense of smell, quality of life, and symptom score |
| **Notes** |  |

Risk of Bias

| **Bias** | **Authors judgment** | **Support for judgment** |
| --- | --- | --- |
| Random sequence generation (selection bias) | Low Risk | Patients block randomization was performed by the institutional center of clinical research. |
| Allocation concealment (selection bias) | Low risk | The information of patients’ allocation was stored in sealed envelopes. |
| Blinding of participants and personnel (performance bias) | Low risk | The clinical evaluation was carried out by ENT specialists  without the knowledge about patients’ allocation to aspirin  or placebo treatment. Study medication was produced by the institutional pharmacy. Placebo medication consisted of lactose, magnesium stearate, cellulose powder and microcrystalline cellulose. |
| Blinding of outcome assessment (detection bias) | Low risk | Study participants and investigators remained blinded to study drug until the study was completed. |
| Incomplete outcome data (attrition bias) | Low risk | Withdrawal rate was considerate at 55% but missingness not related to its true value. |
| Selective reporting (reporting bias) | Low risk | All collected data was reported |

**Swierczynska-Krepa, 2014**

| **Methods** | Design: Pilot, double blind, randomized placebo-controlled study  Setting: Bielsko-Biala and Krakow, Poland |
| --- | --- |
| **Participants** | Participants: n = 34 patients (Aspirin induced asthma (AIA): 20 (12 intervention, 8 placebo), Aspirin tolerant asthma (ATA): 14 (6 intervention, 8 placebo)  Baseline characteristics: mean age in AIA group 46 ± 19 years old, mean age in ATA group 49.5 ± 15.0 years old. Gender distribution (female/male ratio): AIA group 15/5, ATA group 9/5.  Asthma duration (y): AIA group 7 ± 7, ATA group 8.4 ± 9  Aspirin Hypersensitivity duration (y): AIA group 8 ± 8.5, ATA group N/A.  Rhinitis duration (y): AIA group 13.5 ± 12, ATA group 13.5 ± 15  Nasal polyposis duration (y): AIA group 8 ± 9, ATA group 4 ± 13  Systemic steroids (daily dose in ug): AIA group 800 ± 600, ATA group 800 ± 400  Nasal corticosteroids (daily dose in ug): AIA group 100 ± 100, ATA group 100 ± 28  FEV1 (% predicted): AIA group 88.7 ± 17.8, ATA group 92.5 ± 30.9  SNOT20: AIA group 1.9 ± 1, ATA group 1.3 ± 0.7  ACQ: AIA group 1.3 ± 1.4, ATA group 0.9 ± 1  Serum IgE (IU/mL): AIA group 66.7 ± 111.9, ATA group 107.5 ± 90.2  Inclusion criteria:   1. Age 18 to 65 years old, 2) diagnosed asthma, 3) rhinosinusitis with nasal polyps evidenced by medical records, endoscopic findings, and/or CT findings, 4) a prior history of reaction to aspirin or other NSAIDs in the AIA group, 5) negative history of aspirin hypersensitivity and negative aspirin challenge in the ATA group, 6) positive aspirin challenge test: appearance of clinical symptoms and decrease ≤ 20% FEV1.   Exclusion criteria: FEV1 < 70% in spirometry, history of anaphylactic reaction, autoimmune diseases, severe diseases of the heart, digestive, urinary or neurologic system, neoplasm, pregnancy. |
| **Interventions** | Intervention:   - Run-in period: patients underwent a single-blind, 2-day, placebo-controlled oral aspirin challenge to establish the minimum dose of aspirin that provoked symptoms or to rule out aspirin hypersensitivity.   Patients were then randomized to receive either aspirin desensitization or placebo in both the AIA and ATA group.   - Acute desensitization: Patients in the AIA-ASA group received the maximum dose they had tolerated during the aspirin challenge. If the patient remained asymptomatic the dose was incremented every 1.5 hours until reaching a daily cumulative dose of 624 mg/day. If the patient presented symptoms or FEV1 decreased 20% or more, the desensitization was started the next day at the dose that provoked the reaction the day before. This was continued until reaching 624 mg/day without any reactions. - Chronic desensitization: initiated when 624 mg/day were administered and was continued for 6 months.   Other medications: standard asthma medications for rhinosinusitis. Leukotriene modifiers, omalizumab and immunotherapy was not allowed during the study. |
| **Outcomes** | Primary outcomes: clinical efficacy of AD (changes in scores), PNIF, FEV1, PEF values, reduction in corticosteroid doses monthly.  Secondary outcomes: Levels or urinary leukotriene E4 and stable plasma prostaglandin (PG) D_2_ metabolite 9 alpha, 11ß-PGF2 at baseline and at months 1,3,5,6. |
| **Notes** |  |

***Risk of bias***

| **Bias** | **Authors judgment** | **Support for judgment** |
| --- | --- | --- |
| Random sequence generation (selection bias) | Low risk | Patients were randomly assigned using random number tables. No significant difference in baseline characteristics. |
| Allocation concealment (selection bias) | Unclear risk | No information |
| Blinding of participants and personnel (performance bias) | Low risk | Lactose capsules prepared by hospital pharmacy looked identical to aspirin |
| Blinding of outcome assessment (detection bias) | Low risk | Study participants and investigators remained blinded until the study was completed |
| Incomplete outcome data (attrition bias) | High risk | Attrition rate 17.4%; missingness probably due to its true value. |
| Selective reporting (reporting bias) | Low risk | All collected data was reported |
